# Characteristics, Influence, Prevention, and Control Measures of the Mpox Infodemic: Scoping Review of Infodemiology Studies

**DOI:** 10.2196/54874

**Published:** 2024-08-30

**Authors:** XiangYu Yan, Zhuo Li, Chunxia Cao, Longxin Huang, Yongjie Li, Xiangbin Meng, Bo Zhang, Maohe Yu, Tian Huang, Jiancheng Chen, Wei Li, Linhui Hao, Dongsheng Huang, Bin Yi, Ming Zhang, Shun Zha, Haijun Yang, Jian Yao, Pengjiang Qian, Chun Kai Leung, Haojun Fan, Pei Jiang, Tiejun Shui

**Affiliations:** 1 School of Disaster and Emergency Medicine Tianjin University Tianjin China; 2 Department of Epidemiology and Biostatistics School of Public Health Jilin University Changchun China; 3 Department of Clinical Medicine North Sichuan Medical College Nanchong China; 4 School of Basic Medical Sciences Peking University Beijing China; 5 Department of Cardiology The First Hospital of Hebei Medical University Shijiazhuang China; 6 School of Environmental Science and Engineering Hainan University Haikou China; 7 Tianjin Center for Disease Control and Prevention Tianjin China; 8 Yunnan Center for Disease Control and Prevention Kunming China; 9 Xiamen Peiyang BCI & Smart Health Innovation Research Institution Xiamen China; 10 Yunnan Provincial Hospital of Traditional Chinese Medicine Kunming China; 11 Baoshan Prefecture Center for Disease Control and Prevention Baoshan China; 12 Lincang Prefecture Center for Disease Control and Prevention Lincang China; 13 Department of Radiation Oncology The Third Affiliated Hospital of Kunming Medical University & Tumor Hospital of Yunnan Province Kunming China; 14 Yan'An Hospital of Kunming City Kunming China; 15 School of Artificial Intelligence and Computer Science Jiangnan University Wuxi China; 16 Department of Public and International Affairs City University of Hong Kong Hong Kong China; 17 Fairbank Center for Chinese Studies Harvard University Cambridge, MA United States; 18 Department of Public Health North Sichuan Medical College Nanchong China

**Keywords:** mpox, infodemic, infodemiology, information search volume, content topic, digital health

## Abstract

**Background:**

The mpox pandemic has caused widespread public concern around the world. The spread of misinformation through the internet and social media could lead to an infodemic that poses challenges to mpox control.

**Objective:**

This review aims to summarize mpox-related infodemiology studies to determine the characteristics, influence, prevention, and control measures of the mpox infodemic and propose prospects for future research.

**Methods:**

The scoping review was conducted based on a structured 5-step methodological framework. A comprehensive search for mpox-related infodemiology studies was performed using PubMed, Web of Science, Embase, and Scopus, with searches completed by April 30, 2024. After study selection and data extraction, the main topics of the mpox infodemic were categorized and summarized in 4 aspects, including a trend analysis of online information search volume, content topics of mpox-related online posts and comments, emotional and sentiment characteristics of online content, and prevention and control measures for the mpox infodemic.

**Results:**

A total of 1607 articles were retrieved from the databases according to the keywords, and 61 studies were included in the final analysis. After the World Health Organization’s declaration of an mpox public health emergency of international concern in July 2022, the number of related studies began growing rapidly. Google was the most widely used search engine platform (9/61, 15%), and Twitter was the most used social media app (32/61, 52%) for researchers. Researchers from 33 countries were concerned about mpox infodemic–related topics. Among them, the top 3 countries for article publication were the United States (27 studies), India (9 studies), and the United Kingdom (7 studies). Studies of online information search trends showed that mpox-related online search volume skyrocketed at the beginning of the mpox outbreak, especially when the World Health Organization provided important declarations. There was a large amount of misinformation with negative sentiment and discriminatory and hostile content against gay, bisexual, and other men who have sex with men. Given the characteristics of the mpox infodemic, the studies provided several positive prevention and control measures, including the timely and active publishing of professional, high-quality, and easy-to-understand information online; strengthening surveillance and early warning for the infodemic based on internet data; and taking measures to protect key populations from the harm of the mpox infodemic.

**Conclusions:**

This comprehensive summary of evidence from previous mpox infodemiology studies is valuable for understanding the characteristics of the mpox infodemic and for formulating prevention and control measures. It is essential for researchers and policy makers to establish prediction and early warning approaches and targeted intervention methods for dealing with the mpox infodemic in the future.

## Introduction

### Background

Mpox (formerly known as monkeypox) is a zoonotic infectious disease caused by infection with the mpox virus (MPXV) [1]. Clinical manifestations primarily involve fever, skin rash, and lymph node enlargement. The rash is predominantly on the face and limbs, displaying a centrifugal distribution pattern [2]. The main hosts for the MPXV are rodents and primates [3]. Mpox can be transmitted from animal to human and from human to human [4]. Previous studies indicated a degree of cross-protection against the MPXV with the smallpox vaccine, and therefore, the unvaccinated population is generally susceptible [5]. The MPXV belongs to the genus *Orthopoxvirus*, a double-stranded linear DNA virus that is divided into a West African clade and a Congo Basin clade, which is faster spreading and more lethal [6,7]. The first human case of mpox was identified in the Democratic Republic of the Congo in 1970 [8]. Historically, mpox was mainly endemic in West and Central Africa. Reports of mpox outside Africa were rare and primarily involved imported cases or were associated with animals imported from Africa [9]. Since May 2022, numerous mpox cases without travel history to Africa have emerged in multiple countries outside Africa, resulting in international concern as an abrupt public health event [10]. A public health emergency of international concern (PHEIC) was proclaimed for the spreading mpox outbreak on July 23, 2022, by the World Health Organization (WHO) [11]. Most cases involved adults, with most transmission occurring among men who have sex with men (MSM), and the rash is more common in the genital and anal regions [12,13].

In the era of information explosion on the internet, emerging infectious diseases could not only affect human health through human-to-human transmission in the real world but also cause infodemics through the spread of misinformation and rumors on the internet [14]. With the quantity and speed of information rapidly growing, it is challenging for the public to distinguish the true health information and misinformation, which leads to the emergence of an infodemic [15,16]. The lessons we learned from the COVID-19 pandemic revealed the importance of infodemic prevention and control [17]. During the COVID-19 pandemic, incorrect information and rumors distorted the public’s understanding of the pandemic, causing more anxiety and panic, affecting people’s behavior and adherence to public health indications, which posed a significant challenge to disease control and public health. Incorrect recommendations for treatment methods, prevention, or behaviors could potentially be harmful and worsen the current pandemic [18]. Tedros Adhanom Ghebreyesus, WHO’s director-general, has said that “We’re not just fighting a pandemic; we’re fighting an infodemic” [19].

Similar to the COVID-19 pandemic, the outbreak of mpox in 2022 led to widespread searches and sharing of mpox-related information on search engines and social media platforms [20,21]. Several studies based on social media, such as Twitter, Facebook, and TikTok, have found that expressions of negative sentiments, attitudes toward vaccines, homophobia and racism, and various comments about symptoms and prevention methods are widely discussed on social media [22-25]. This has sounded the alarm of the emergence of a new infodemic.

Infodemiology studies the distribution and determining factors of health information and misinformation on the internet and among populations, which has significant practical applications in the research of infodemic identification and control [26]. It involves analyzing, monitoring, and predicting certain diseases’ outbreaks and epidemics and tracking health-related behaviors in populations and other areas based on online data [27]. Infodemiology provides an important perspective and support for exploring the characteristics of infodemics and can contribute to decision-making in digital health to cope with the ongoing infodemic. At present, there is an urgent need to comprehensively assess the situation and impact of the mpox infodemic to offer recommendations for the prevention and control of mpox through online platforms. However, there is a lack of reviews on this particular topic.

### Objective

The aim of this review is to comprehensively summarize mpox-related infodemiology studies in the following aspects and propose prospects for future research: (1) the characteristics of internet search activities and infodemic trends during mpox outbreaks, (2) the views and opinions of social media users and the psychological impact of the mpox infodemic, and (3) researchers’ suggestions on prevention and control measures for the mpox infodemic.

## Methods

### Study Design and Literature Search

The structured 5-step methodological framework by Arksey and O’Malley [28] was used to form this scoping review, including (1) delineating the research query; (2) pinpointing pertinent studies; (3) selecting studies; (4) extracting and charting data; and (5) collating, synthesizing, and presenting the results.

A total of 4 electronic literature databases, including PubMed, Web of Science, Embase, and Scopus were searched comprehensively till April 30, 2024, to avoid missing important studies. The following four parts of keywords were combined as the entry terms when searching the 4 databases: (1) mpox-related keywords: “monkeypox” or “mpox”; (2) infodemic-related keywords: “infodemic” or “infodemiology” or “infosurveillance” or “infoveillance”; (3) website and search engine–related keywords: “internet” or “web” or “website” or “online” or “search engine” or “search volume”; (4) media-related keywords: “media” or “medium” or “social app” or “social application” or “mobile app” or “mobile application” or “software” or “blog” or “blogging” or “forum”; and (5) combinations of #Part 1 AND (#Part 2 OR #Part 3 OR #Part 4).

As these databases collectively included articles from representative preprint repositories, including arXiv, bioRxiv, medRxiv, and Research Square, the searching process could effectively capture cutting-edge studies and articles that had not been peer reviewed.

### Study Selection

A study selection procedure was conducted after eliminating duplicates. The inclusion and exclusion criteria of literatures are shown in Textbox 1. A total of 2 reviewers (XY and ZL) screened the literature and documents independently. Disagreements were resolved by discussion with a third reviewer (PJ or L Huang).

**Inclusion and exclusion criteria of study selection.**

**Inclusion criteria**

- Article type
  Original research
- Language
  Provide at least a clear abstract or summary in English
- Full text
  The full text of the study could be found
- Content of the article
  Focused on mpox
  Analysis of internet information
  Focused on online information trends analysis, content analysis, or prevention and control measures for the mpox infodemic

**Exclusion criteria**

- Article type
  Nonoriginal research, including review (because the original studies in the review had been included in the study), commentary, and short conference abstract
- Language
  Both the abstract or summary and main text were not in English
- Full text
  Only had short abstract or summary, without full text
- Content of the article
  Not relevant to mpox
  Not relevant to internet information analysis
  Only conducted online surveys through the internet, and the content was not relevant to the infodemic

### Data Extraction

After a thorough reading of full text, the following information of the selected studies were extracted: (1) article title, (2) journal name, (3) authors and countries, (4) publication date, (5) search engine or social media, and (6) main findings. For articles that mainly focused on the online information search volume or content analysis of social media text, quality assessment was conducted based on the following four criteria: (1) specified the search engine or social media application; (2) clearly reported the content source of the online information, such as Google Trends, Baidu Index, posts, comments, tweets, and videos; (3) clearly reported the period and the amount of data acquired; and (4) reported the statistical analysis method and process. If the abovementioned information could not be fully identified in the article, we considered the article to be incomplete and of low quality, which would be excluded. A total of 2 reviewers (XY and ZL) extracted data and conducted quality assessment, respectively, and reconciled their data records and quality assessment results after this process. A third reviewer (TS) was consulted to make the final decision if there were disagreements.

### Data Synthesis

The number of publications per month were calculated, and the chronological trend of publications were described. On the basis of the authors’ countries, the geographical distribution of the publications was described using a map. According to the main findings extracted using the abovementioned process, the main focuses of the mpox infodemic were summarized and categorized into 4 aspects, including trends of online information search volume, content topics of mpox-related online posts and comments, emotional and sentiment characteristics of online content, and prevention and control measures for the mpox infodemic. Statistical analyses were performed using SPSS (version 21.0; IBM Corp). Mapping was performed with ArcGIS (version 10.0; ESIR).

## Results

### General Characteristics of the Included Studies

**Figure 1:**
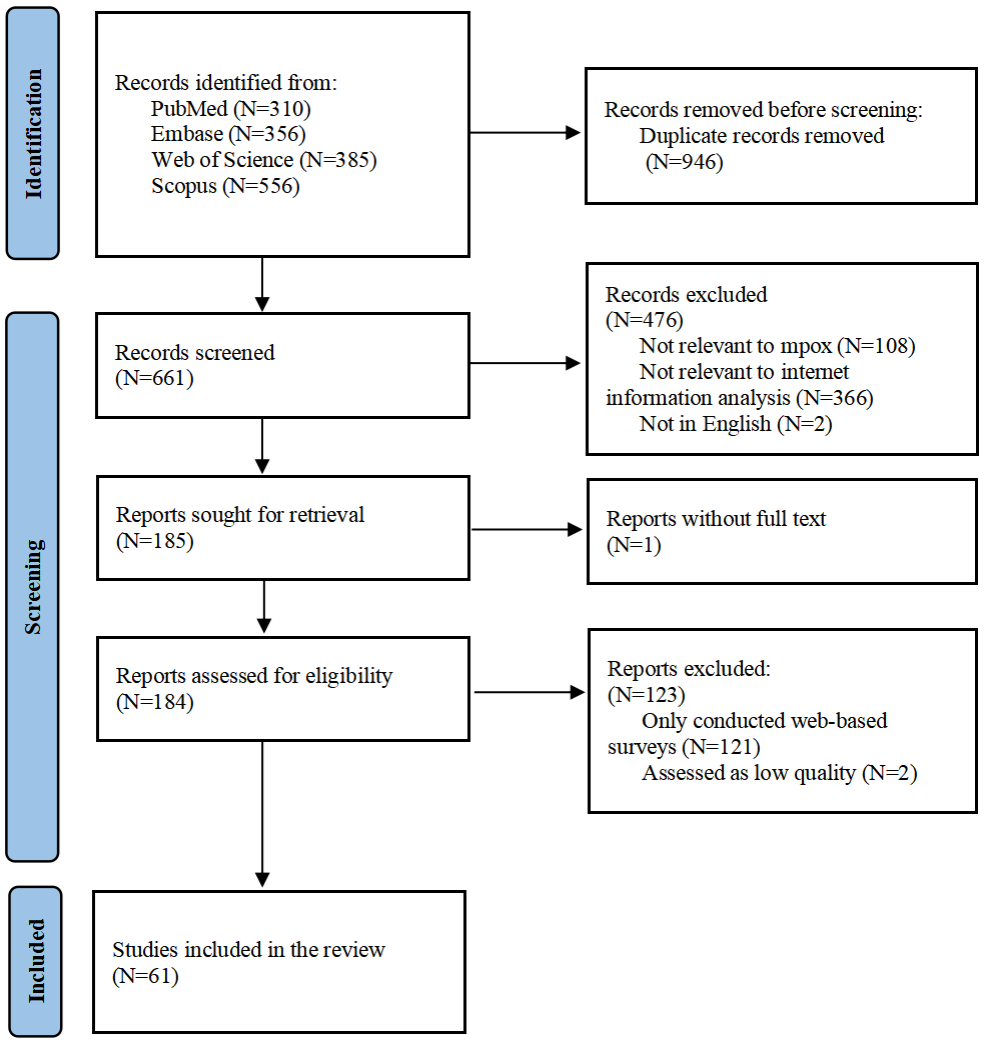
Study selection flowchart of mpox infodemiology studies by April 2024.

A total of 1607 articles were retrieved from the databases according to keywords. After the removal of duplicates and study selection based on exclusion criteria, 61 studies were included in the final analysis (Figure 1). The first study reporting online social media’s information of mpox was published in November 2019, which focused on the media reporting of mpox in Nigeria. After that, few studies focused on mpox-related online information or the infodemic until May 2022, when mpox cases emerged in multiple countries outside Africa. With WHO’s declaration of an mpox PHEIC, the number of related studies began growing rapidly (Multimedia Appendix 1).

Some popular search engines and social media applications had become the main research platforms and tools for researchers, such as Google, Baidu, TikTok, Twitter, Facebook, and Reddit. Google was the most widely used search engine platform (9/61, 15% studies) for researchers to describe online information search volume. For social media applications, Twitter had become the most used platform for researchers (32/61, 52% studies; Multimedia Appendix 1).

**Figure 2:**
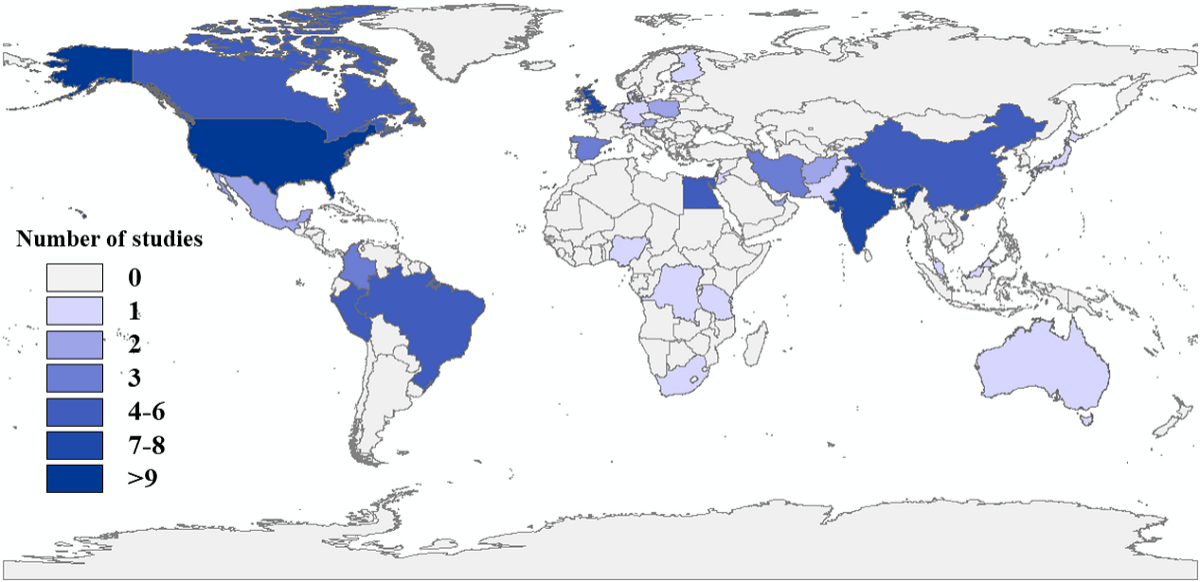
Global geographical distribution of mpox infodemiology studies from November 2019 to April 2024.

Researchers from 33 countries were concerned about mpox infodemic–related topics. The top 3 countries that published a relatively large number of mpox infodemic articles were the United States (27 studies), India (9 studies), and the United Kingdom (7 studies; Figure 2).

### Trends in Online Information Search Volume

Public concern about mpox first showed up in web-based search activities. As an important web search engine, Google provides a useful tool for quantifying web-based search activities named Google Trends [29]. On the basis of Google Trends, Du et al [30] showed that after declaring the outbreak of mpox as a PHEIC, the global web-based search volume of mpox-related keywords increased by 42.85%, and the increase trends occurred among 62 countries or territories, especially for Brazil, the United States, and Indonesia. According to the comparison of Google Trends in mpox endemic countries (eg, Nigeria and Ghana) and nonendemic countries (eg, Spain and Germany) in 2022, Shepherd et al [31] observed a common pattern in most of these countries: online health information–seeking behavior was triggered by the first human mpox case and quickly peaked; however, this increasing trend only lasted for a short time. In addition, compared to the consistently low online search volume for COVID-19 information from mid-May to mid-August 2022, public interest in mpox showed significant fluctuations, with 2 peaks. These peaks occurred when the WHO declared that mpox could be transmitted through sex and when the WHO declared mpox a PHEIC [32]. However, the concerns of COVID-19 still exceeded mpox in most low- and middle-income countries in this period [32]. One study evaluated the search for specific ophthalmic symptoms of mpox in detail (eg, mpox eye), of which Canada, the United States, and the United Kingdom had the top 3 highest search rates [20].

Baidu is a commonly used search engine for Chinese internet users [33]. Similar to the Google Trends tool, the Baidu Index provides the weighted values of keywords search frequencies through the Baidu search engine, which can dynamically reflect the search trend of keywords and public attention [34]. On the basis of the Baidu Index, a study conducted in China showed that the searching trend of mpox in China fluctuated with key events, and the peaks occurred at the following time points: (1) WHO issued the mpox outbreak alert in May 2022; (2) first imported cases of mpox were reported in South Korea and Taiwan, China, in June 2022; (3) WHO declared the mpox outbreak as a PHEIC in July 2022; and (4) Hong Kong and Chongqing, China, reported the first imported cases of mpox [35].

In addition to internet search engines, several studies have revealed the volume of mpox information on other social media. Kato et al [36] investigated media coverage of mpox-related information on 14 media websites in Japan. The volume trends could be divided into 3 periods, including an initial phase between May 19 and May 22, 2022, when the outbreak occurred in the United States and Europe; a judgment period between June 15 and June 16 when the reports on the websites mainly discussed whether mpox should be declared a PHEIC by WHO; and a surge period between June 22 and June 29 when the outbreak began in Asia. Similar to the Google Trends studies, a global infodemiology study based on searches for sexually transmitted infections on YouTube showed a sudden increase began from the WHO’s declaration for mpox’s spread through sex, and another peak occurred after the WHO’s declaration of the PHEIC [21]. In addition, through sampling the text of 105,913,149 tweets, Brian et al [37] detected the number of mpox-related and homophobic tweets from January to October 2022. This study showed a peak of both trends in July and indicated the polarizing trend in mpox-related homophobic and online hate against gay, bisexual, and other MSM (GBMSM) populations, which were catalyzed by the global mpox crisis [37].

### Content Topics of Mpox-Related Online Posts and Comments

**Figure 3:**
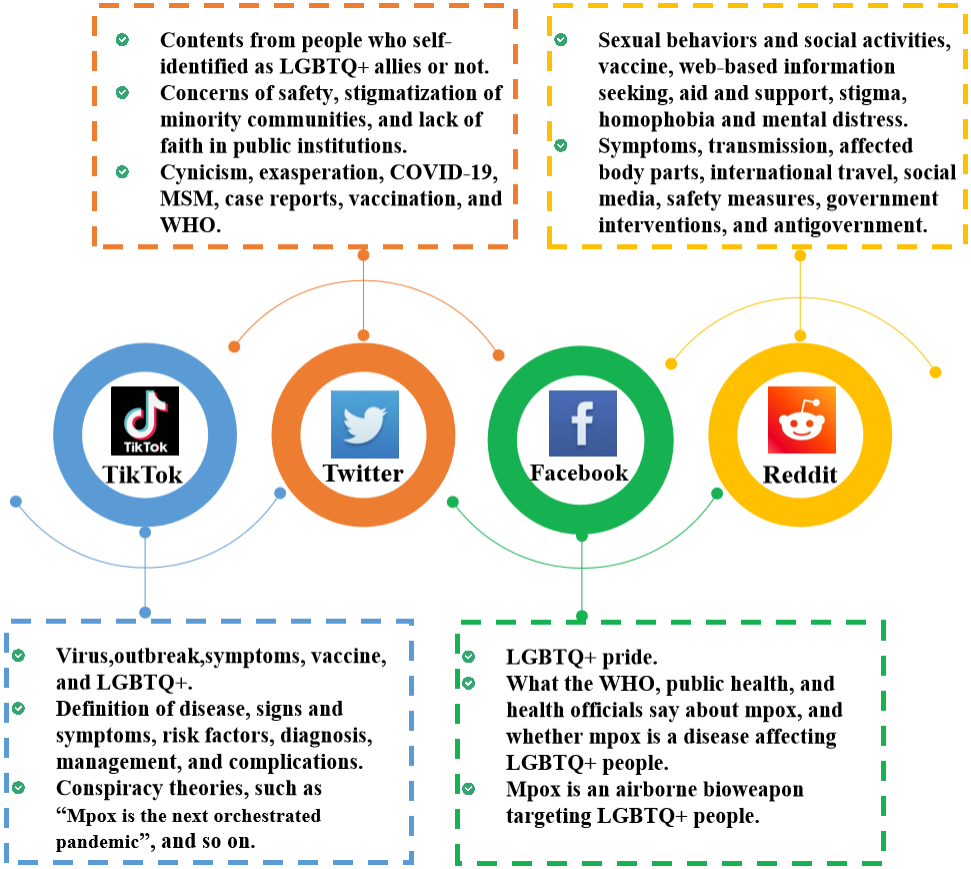
Content topics of mpox-related posts and comments on 4 popular social media from November 2019 to April 2024. LGBTQ+: lesbian, gay, bisexual, transgender, and queer; MSM: men who have sex with men; WHO: World Health Organization.

The contents of the public’s concern on online social media were the main focus of the previous infodemiology studies. Public opinions on the 4 popular social media were widely discussed, including TikTok, Twitter, Facebook, and Reddit (Figure 3).

TikTok is a short video mobile platform that allows users to create videos lasting between 15 seconds and 10 minutes [38]. Zenone and Caulfield [39] identified 17.7% (153/864) videos with conspiracy theories or themes related to the mpox outbreak on TikTok, which was classified into 11 categories (eg, “Mpox is the next orchestrated pandemic,” “Mpox was introduced to administer vaccines,” and “Bill Gates is involved in the mpox outbreak”). By analyzing 100 English videos, Ji-Xu et al [25] found that the video types were mainly educational content (82%) and patient experience (72%); hashtag categories included virus (26%), outbreak (17%), symptoms (15%), vaccine (13%), and lesbian, gay, bisexual, transgender, and queer (LGBTQ+; 12%). For the quality of information, videos uploaded by doctors and science communicators had better information quality and fewer misleading statements compared with other users; however, they had a lower mean number of views and likes [25,40]. In addition, there was inconsistency in the proportion of videos containing misleading statements. Ortiz-Martínez Y and Ortiz-Martínez HM [41] found that only 1% of the most popular videos displayed misinformation, while Ji-Xu et al [25] found that 5 (14%) of the 36 physician videos and 21 (33%) of 64 nonphysician videos had misleading statements. Shi et al [40] studied 85 videos containing public-directed educational information about mpox and found that 33.33% of them were highlighted in clinical practice guidelines (ie, definition of disease, signs and symptoms, risk factors, diagnosis, management, and complications); however, these contents showed incomplete and inaccurate information frequently.

For posts on Twitter, the discussion of mpox-related contents was more extensive. Ortiz-Martínez et al [42] manually checked the top 100 tweets from late May 2022 and found that 52% of tweets contained misinformation and 20% were humorous or nonserious, with the most common misinformation claiming the mpox outbreak was a conspiracy. Edinger et al [43] analyzed 230,163 tweets from May 7, 2022, to July 23, 2022, and identified seven content clusters using the Sentence Bidirectional Encoder Representations from Transformers: (1) cynicism, (2) exasperation, (3) COVID-19, (4) MSM, (5) case reports, (6) vaccination, and (7) WHO. A study conducted by Ng et al [44] focused on topic modeling and manual thematic analysis by using a large data set of >350,000 tweets, which derived 5 topics into 3 major themes related to the public discourse on the ongoing outbreaks, including concerns of safety, stigmatization of minority communities, and a general lack of faith in public institutions. Farahat et al [45] conducted topic modeling with latent Dirichlet allocation (LDA) using Twitter data collected from May 22, 2022, to August 5, 2022, and identified 12 topics, including the possibility of mpox being pandemic, mpox origin, gay community, spread, preventive measures, declaration of the outbreak, work, the need for mpox vaccinations, confirmed cases, mpox surfacing amid the COVID-19 pandemic, sexually transmitted, and beliefs about mpox. Another study showed that the uncertainty expressions about mpox outbreak turned to be less common among Twitter users in the week after WHO officially declared the mpox PHEIC than the week before WHO’s declaration, which indicated that the official public health messaging could correct misperceptions and false narratives [46].

Most discussions on mpox were centered on the gay and other sexual minority groups. Many people have commented that mpox will be spread through their sexual behaviors. Homophobic and discriminatory information could cause stigma among this population. Cooper et al [22] examined 858,581 tweets from May 1, 2022, to July 23, 2022. Topic modeling by LDA identified unique themes among people who self-identified as LGBTQ+ allies or not [22]. Self-identified allies discussed 6 main topics, including informing prevention for mpox infection, geographic reporting of mpox cases, sharing of mpox vaccine information, lessons from mpox and other epidemics, checking hetero-privilege about mpox transmission, and pandemic exasperation about vaccination [22]. As for tweets from non–self-identifying allies or advocates, 9 topics were identified, including growing fear about mpox pandemic, geographic reporting of mpox cases, sexual transmission of mpox, learning from smallpox vaccine for mpox, anger about mpox, concerns about mpox as a global health emergency, global health response and mpox vaccine, declaration of mpox as a global health emergency, and surprise at mpox spread outside Africa [22]. Drawing data from Facebook and Twitter, Movahedi et al [47] used LDA to explore country-level variations in topics related to mpox and two-spirit, lesbian, gay, bisexual, transgender, queer or questioning, intersex, and asexual (2SLGBTQIAP+). They found that a total of 10 different topics were constructed from posts, and 8 were aimed at stigmatizing the 2SLGBTQIAP+ community. For posts on Reddit, Hong [48] used the method of inductive coding approach to analyze 755 Reddit posts that were popular among GBMSM and summarized four important themes: (1) changes in sexual behaviors and social activities; (2) mpox vaccine attitude, uptake, and hesitancy; (3) online information seeking and mutual aid and support; and (4) perceived and experienced stigma as well as homophobia and mental distress. Anoop and Sreelakshmi [49] used LDA and human-in-the-loop qualitative analysis to analyze 289,073 Reddit comments from June 1, 2022, to August 5, 2022. A total of 10 topics were obtained, which included mpox symptoms, mpox transmission, affected body parts, international travel, social media, safety measures, government interventions, mpox vaccination, antigovernment, and homophobia [49].

### Emotional and Sentiment Characteristics of Online Content

Emotional and sentiment analysis was also a focus of previous studies. The following methods were used: the National Research Council emotion lexicon [45]; Valence Aware Dictionary and Entiment Reasoner methodology [23]; convolution neural network-long short-term memory network hybrid-based model [50]; certain software packages, such as TextBlob [45], pysentimiento package, and CamemBERT [47,51]; SentiStrength library; and text2emotion library [22].

Although the proportion of different emotional and sentiment types varied in different studies, negative ones accounted for a significant proportion. In a study focusing on mpox tweets in Latin America, which included 55,009 Spanish-language tweets collected between May 31, 2022, and October 31, 2022, the number of negative tweets published by users was much more than positive ones during the 23 weeks analyzed, representing 91.57% [52]. The first public data set of mpox tweets was presented and released by Thakur [23]. This study conducted sentiment analysis of the Twitter posts related to the mpox outbreak from May 7, 2022, to 23 July 2022, revealing a majority neutral sentiment (62.75%) toward mpox, followed by “negative” (20.91%) and “positive” (16.34%) sentiments [23]. Cooper et al [22] analyzed >850,000 English-language tweets from May 1, 2022, to July 23, 2022. They found that 46.5% of tweets were classified as neutral sentiment scores, 39% were negative, and 14.5% were positive. Farahat et al [45] analyzed a collection of >5000 mpox-related tweets from May 22, 2022, to August 5, 2022, and found that 48% of tweets were neutral sentiment, 15% were negative, and 37% were positive. Iparraguirre-Villanueva et al [50] analyzed 84,018 Spanish tweets obtained from Peru in September 2022 and found that 45.42% of people expressed neither positive nor negative opinions, 19.45% expressed negative and fearful feelings, while 35.13% of the opinions regarding the MPXV were positive.

Furthermore, several studies reported that the proportion of posts with positive sentiment has exceeded negative and neutral ones. Anoop and Sreelakshmi [49] analyzed 289,073 Reddit comments from June 1, 2022, to August 5, 2022, and found that nearly 35% of these comments were positive, followed by negative sentiment (34%) and neutral ones (31%). In addition, it seemed that vaccination and immunization-related posts showed more positive sentiment. Rajkhowa et al [51] analyzed 149,133 tweets related to mpox vaccination and immunization from May 1, 2022, to September 23, 2022, and found that the proportion of tweets with positive sentiment was 37.05%, higher than that of negative sentiment (29.89%) and neutral sentiment (33.06%).

A total of 2 articles reported the sentiment difference across different user characteristics. Cooper et al [22] compared sentiment characteristics between posts of self-identified LGBTQ+ allies and nonallies and indicated that most sentiments and emotions values, including happy, surprise, fear, and sadness, except for anger, showed significant differences between the 2 groups. Movahedi et al [47] reported a geographic difference in sentiment intensity among different countries by analyzing 125,424 Twitter and Facebook posts related to mpox and the 2SLGBTQIAP+ community, of which English posts had higher negative and lower neutral and positive sentiment intensities than Spanish and French posts, and Spanish posts had higher negative and lower positive sentiment intensities than French ones.

In addition, Hong [48] analyzed 755 Reddit posts in several subreddits that were popular among GBMSM and showed obvious anxiety emotion about the outbreak and mpox-related stigma and discrimination among them. These posts also showed their desire and confidence in mpox vaccination in preventing themselves from infection and their concerns about getting vaccinated, such as their eligibility and concerns about vaccine side effects and effectiveness.

Another emotional expression of social media users was pandemic fatigue, which was characterized by people’s reduced compliance with public health directives over time [53]. Through analyzing 1,484,042 social media posts from multiple social media platforms between June 23, 2022, and September 21, 2022, White et al [53] reported several emotional themes of pandemic fatigue, including anger, apathy or disinterest toward preventive measures, and overwhelmed with multiple or sustained emergencies.

### Prevention and Control Measures for the Mpox Infodemic

#### Overview

As mentioned in the previous sections, due to the public’s lack of understanding of mpox-related knowledge when the mpox outbreak occurred, false news and misinformation filled online platforms and social media, resulting in negative emotions such as fear and panic about mpox and discrimination against mpox key populations such as GBMSM. The mpox infodemic could worsen the actual epidemic situation and conceal the voices of health experts and trustful information [24]. A similar serious infodemic also occurred during the COVID-19 pandemic, especially in the early stage [54]. Therefore, it is essential to learn the lessons of the COVID-19 pandemic and take early prevention and control measures [32]. To prevent the mpox infodemic from becoming worse, previous infodemiology studies on mpox put forward several digital health suggestions, which could be summarized as the following 3 aspects. Detailed suggestions can be seen in Table 1.

**Table 1:** Main suggestions on mpox prevention and control in infodemiology studies from November 2019 to April 2024.

References	Social media and platforms	Main suggestions on mpox infodemic prevention and control
Zenone and Caulfield [39], 2022	TikTok	Public health experts may consider greater attention to and investment in monitoring the online environment.
Ortiz-Martínez et al [42], 2022	Twitter	Offering accurate and reliable information via social media platforms can help combat infodemics, misinformation, and rumors in the current mpox outbreak. Real-time mpox surveillance on social media.
Ng et al [44], 2022	Twitter	Authorities should actively counter misinformation, address stigmatizing portrayals, and rebuild the public’s faith in institutions. Primary care practitioners should also be equipped with the tools and knowledge to provide culturally sensitive interventions for LGBTQ+^a^ populations. Public health institutions could also engage respectable key opinion leaders in the LGBTQ+ community to provide the correct public messaging on mpox transmission, signs and symptoms, available help, and prophylaxis.
Martins-Filho [21], 2022	YouTube	Implementing devices that monitor YouTube content in real time, preventing the spread of false and discriminatory news about mpox.
Martins-Filho et al [32], 2022	Google	Lessons learned during the COVID-19 pandemic that information spread was driven by the interaction paradigm imposed by specific social media and by the interaction patterns of users engaged with the topic must be put into practice in mpox infodemic control.
Frost and Baldwin [55], 2022	Google	The need for readability to be taken into account when publishing online resources to ensure simpler information is made available on this topic.
Farahat et al [24], 2022	Multiplatforms	Raising public awareness and preventing stigma through cooperation with stakeholders. Social media platforms need to provide accurate official news. One health approach should be established and prioritized.
Hong [48], 2023	Reddit	Understanding gay, bisexual, and men who have sex with men’s concerns and prioritizing their needs. Addressing the historical medical mistrust and promote vaccine education. To disseminate vaccine information and knowledge through popular opinion leaders. Using social media as a platform for intervention delivery.
Anoop and Sreelakshmi [49], 2023	Reddit	The user-generated responses collected and analyzed from social media and news aggregators identify outbreak clusters and hate groups. To discover public sentiment toward the measures taken by governments and public health organizations so that they can change their strategies and prioritize the action items.
Ji-Xu et al [25], 2023	TikTok	Develop and validate specific quality assessment tools to evaluate video quality on social media. Physician creators posting mpox content on TikTok should consider including a discussion of treatments and using hashtags selectively to increase user engagement.
Shi et al [40], 2023	TikTok	Producing higher quality and richer videos. Collaboration with influencers can promote health communication messaging, increase disease awareness, and enhance audience engagement.
Ortiz-Martínez Y and Ortiz-Martínez HM [41], 2023	TikTok	Leveraging the influence of health care professionals and organizations on TikTok.
Basch et al [56], 2023	TikTok	It is feasible to create engaging videos that mention prevention measures, such as vaccines, disinfection, and safe sex.
Cooper et al [22], 2023	Twitter	Continued analysis of social media posts could allow public health officials to monitor public opinion on the next outbreak and spread relevant warnings and preventive measures to a wider audience sooner. To determine groups of patients requiring targeted disease education based on the amount of misinformation disseminated. Understanding how various demographic groups discuss a disease’s symptoms or spread could allow targeted preventions such as vaccines to be mobilized to those populations quicker.
Edinger et al [43], 2023	Twitter	Social media is monitored in real time to detect the dissemination of information that does not align with public health objectives. Wide and timely dissemination of important public health information. Gain insight into potential messaging campaigns that go beyond stigma and stereotypes. Greater digital surveillance will ensure that information reaches people at a faster rate.
Keum et al [37], 2023	Twitter	Research examining mpox-based stigma and online homophobia in international contexts is required.
Rajkhowa et al [51], 2023	Twitter	Policy makers must prioritize disseminating adequate information to physicians and the general population to ensure optimal vaccination coverage. Various interim guidance on vaccines and immunization for mpox can be adopted. Strengthening the mental health status among the community. Creating communication strategies or hosting workshops focused on building trust and effective communication regarding vaccines and immunization can help minimize vaccine-related fears
Movahedi et al [47], 2023	Twitter and Facebook	Destigmatizing social media through protest, education, and engagement.
Du et al [30], 2023	Google	The time-lag effect of global online search activity on daily new cases was significant. Online search activity could be used as an early indicator of the outbreak of mpox at the global level and in epidemic countries.
Shepherd et al [31], 2023	Google	Publication of first mpox case promotes a surge in online health information seeking; the spike in searching behaviors occurs in a relatively short window. Health bodies and governmental organizations must work to ensure that credible and accurate information is searchable and in place as new disease cases or large news publication events are disclosed to the public. For countries with low internet penetrance, the dissemination of public health–related information (eg, transmission prevention measures) must rely on different traditional infrastructures and media (eg, newspapers and radio).
Zheng et al [35], 2023	Baidu	After the outbreak of an international epidemic, domestic epidemic information should be disclosed in real time, and core information, such as disease characteristics, transmission routes, and immunization prevention, should be compiled as soon as possible to address issues of public concern. The content of online health education should be different at different stages of the epidemic. Before the outbreak of imported epidemics, attention should be paid to the health publicity of immunization prevention of the disease, the harm of the disease, and animals to avoid contact with. Later attention should be paid to the publicity and education of early symptoms and transmission routes of the disease. The health education content should be concise and easy to understand and could be carried out in vivid and interesting ways, such as short videos and cartoons.
Kato et al [36], 2023	14 media websites in Japan	It is essential to provide easy-to-understand information, enabling the media to disseminate basic information on infectious diseases and messages that lead to coping appraisals.
Dsouza et al [57], 2023	Twitter	A system is needed to track the dissemination of unregulated media, especially social media. Each nation needs to strengthen its health system and create comprehensive risk communication strategies and plans to prevent mpox stigma among the LGBTQ+ without jeopardizing their well-being.

^a^LGBTQ+: lesbian, gay, bisexual, transgender, and queer.

#### Timely and Active Publishing of Professional, High-Quality, and Easy-to-Understand Information Online

Health professionals should be organized to publish authoritative, high-quality mpox information in collaboration with online media influencers in advance of widespread misinformation [25,40,41]. The health guidance information should include information on vaccines, early symptoms, modes of transmission, harm of the disease, diagnosis, and treatment recommendations [22,51,56]. Shepherd et al [31] suggested that it is necessary to grasp the short time window of the searching behaviors spike when the government declared the first local mpox case to publish high-quality health information. The content of online health education should be different at different stages of the epidemic: (1) before the outbreak of imported epidemics, attention should be paid to health publicity of immunization prevention of the disease, the harm of the disease, and animals to avoid contact with and (2) later attention should be paid to the publicity and education of early symptoms and transmission routes of the disease [35]. In addition, the professional information published through online platforms and social media should be easy to understand and have high readability, which can be carried out in vivid and interesting ways, such as short videos and cartoons [35,36,55]. For regions with low internet accessibility, more attention for health information publication needed to be paid to traditional media (eg, newspapers and radio) [31].

#### Surveillance and Early Warning of the Infodemic Based on Internet Data

Several studies have suggested that it is necessary to consider greater investment and tool development for real-time surveillance of mpox-related content on social media [21,39,42,43,57]. There are 2 advantages to enhance online surveillance of mpox information. On the one hand, real-time grasp of the situation of fake news and public’s negative emotions on the internet is conducive to timely intervention and preventing misinformation from occupying the mainstream and forming social panic.

On the other hand, a previous study indicated that there was a significant time lag between the increase of online information search volume and the increase of daily new cases [30]. Du et al [30] has indicated that the higher mpox-related global online search volume, the more daily mpox cases would be observed thereafter, especially after a 7-day and 14-day time lag. Therefore, monitoring online mpox information can provide early indicators for early warning of the mpox epidemic in real world.

#### Protection for Key High-Risk Mpox Populations, Such as GBMSM

People who were most affected by the infodemic were the mpox high-risk population such as GBMSM. Online platforms and social media were rife with discrimination, stigma, homophobia, and even hatred against this subpopulation [37,49]. Therefore, to reduce the harm caused by cyber violence, it is essential to actively understand their concerns and needs and counter misinformation and stigmatizing portrayals through education and engagement on online platforms [47,48]. At the health system and policy-making level, comprehensive and well-developed risk communication strategies and plans are needed at the beginning of stigmatized diseases’ epidemic to prevent stigmatization on the population considered vulnerable without blaming the affected [57]. In addition, it is of great value to turn online platforms and social media into fronts of interventions for high-risk populations. Popular opinion leaders in the communities of these populations can contribute to disseminating vaccine information and correct health knowledge [44,48].

## Discussion

### Principal Findings

This was an innovative study that reexplored the global mpox pandemic from the perspective of the infodemic and infodemiology. Before the current global pandemic, the mpox infodemic and its influence received little attention, which was due to mpox being locally endemic in West and Central Africa, with few reports outside Africa before 2022 [9]. West and Central African countries lag behind high-income countries in internet infrastructure and social media development, with relatively low population penetration of the internet and mobile devices. The relatively rapid growth trend of mpox infodemic studies after 2022 indicated that the mpox pandemic has indeed aroused widespread concern and discussion among people around the world and has brought anxiety and panic. This further demonstrates that the global infodemic resulting from a new pandemic of emerging infectious diseases cannot be ignored.

The influence of the mpox infodemic was not only reflected in the rapid increase of the relevant online information search volume and the expression of negative emotions on social media but also some conspiracy theories that contributed to hesitation to take the mpox vaccine. Some assertions led to the idea that mpox was a planned pandemic introduced for power, control, and money because vaccine manufacturers and governments could administer or mandate vaccines worldwide [39]. The wide spread of such views online undermined the credibility of government actions and people’s confidence in mpox vaccines. In addition, high-risk populations such as GBMSM bore a huge impact of the infodemic. They had experienced stigma and discrimination brought by mpox and their identity as GBMSM, which could exacerbate their vaccine hesitancy and deter them from seeking health care services as early as possible [48]. The abovementioned influences of the mpox infodemic could increase the difficulty and reduce the efficiency of mpox prevention and control work.

Another important finding of this study was that we should attach great importance to the guiding role of information release by authoritative institutions and individuals on online public opinion. For instance, the WHO is the international flagship agency for infectious disease prevention and control. The WHO’s declaration of the mpox pandemic as a PHEIC in July 2022 significantly increased the popularity of mpox-related information and discussion on the internet. The studies based on Google Trends and Baidu Index showed that the public’s web-based searching behaviors of mpox-related information peaked after WHO’s PHEIC declaration [32,35]. Similar phenomena were also shown in the increasing information volume on social media platforms such as YouTube and Twitter [21,37]. In addition, our study showed the increasing trends in the number of mpox infodemic–related articles after July 2022, which indicated that researchers were paying more attention to the mpox infodemic after the WHO’s PHEIC declaration. For better prevention and control of the ongoing infodemic, it is vital to make full use of authoritative institutions and opinion leaders to lead the release of correct information on the internet. There were good experiences for us to learn from the control of the COVID-19–related infodemic. During the COVID-19 pandemic, the government’s timely correction of misinformation and refutation of rumors could help curb people’s panic buying [58]. The study by Wang et al [59] also indicated that the opinion leaders played a key role in leading discussions about COVID-19 vaccines on social media. Therefore, when a public health emergency occurs, prevention and control workers should promptly formulate an infodemic response plan and release professional and high-quality information via authoritative departments and social media opinion leaders.

### Perspectives for Future Research

Given the characteristics and limitations of previous explorations of the mpox infodemic, it is essential to involve more digital health approaches to address the new crisis. Researchers can consider the following five aspects to further implement mpox-related infodemiology research and infodemic control.

First, it is essential to establish a comprehensive index system to evaluate the levels of the mpox infodemic based on online information search volume, the proportion of fake news and misinformation, and local mpox case numbers. This system will allow for a quantitative assessment of the risk of the infodemic.

Second, building and optimizing open-source large-scale online information databases can solve the technical bottleneck of data acquisition for researchers and improve research efficiency. Nia et al [60] and Thakur [23] have provided 2 large-scale Twitter data sets for researchers. Large data sets of other social media that are constantly updated are also urgently needed. Importantly, the data acquisition and database building process should comply with internet information and personal privacy security laws, regulations, and ethical requirements, which should also meet the information acquisition stipulations of social media. In addition, to adequately protect privacy and avoid information leakage, the administrators of the open-source databases should pay attention to the removal of personally identifiable information in the internet databases and enter into data security agreements with data users.

Third, it is necessary to carry out a series of comparative studies. It is valuable to compare the infodemic characteristics of countries with different mpox epidemic levels and cultural characteristics. Besides, the comparison of online information search volume and mpox-related content’s topics on online platforms and media of different user groups (ie, Blued and Zank used mainly by MSM population and Twitter and TikTok used commonly by general populations) is helpful to formulate targeted online prevention and control strategies.

Fourth, mpox epidemic prediction and early warning models can be constructed based on information on online platforms and social media. This work can provide a new way to improve the timeliness of response for potential outbreaks of mpox.

Fifth, there is a need to develop targeted digital health intervention measures for the mpox infodemic and conduct effectiveness evaluation research for intervention measures. Previous infodemiology studies mainly focused on the searching trends and content of mpox-related information and only put forward suggestions and prospects for prevention and control measures. There is a lack of research to develop and evaluate the effectiveness of specific and feasible infodemic control measures. Therefore, this area of research needs urgent attention from researchers and policy makers.

### Limitations

There were 2 limitations of this study. First, only articles written in English were included in this study. Articles published in other languages might be missing. Second, our search of selected databases might have excluded publications from nonindexed sources.

### Conclusions

This study comprehensively summarized the characteristics, influence, prevention, and control measures of the global mpox infodemic. The number of related studies began to rise after the WHO’s declaration of an mpox PHEIC, which involved researchers from 33 countries around the world. Popular search engines such as Google and social media such as Twitter became the main research platforms and tools for mpox-related infodemiology studies. The peaks of mpox-related online information search volume occurred at the beginning of outbreaks when the first mpox case in a country was reported and when the WHO provided important declarations, such as the PHEIC. However, the peaks always lasted for a short time window. Through analyzing the content topics of posts and comments on social media, a large amount of misinformation with negative sentiment and discriminatory and hostile content against key populations such as GBMSM was found, which posed significant challenges to the control of the mpox epidemic. Given the significant impact of the global mpox infodemic, positive prevention and control measures for the mpox infodemic are needed, including timely and active publishing of professional, high-quality, and easy-to-understand information online, strengthening surveillance and early warning of the infodemic based on internet data, and taking measures to protect key populations from harm from the mpox infodemic.

## Appendix

Multimedia Appendix 1 Number of mpox infodemiology studies from November 2019 to April 2024 and the commonly used search engines or social media. PHEIC: public health emergency of international concern; WHO: World Health Organization.

Multimedia Appendix 2 PRISMA (Preferred Reporting Items for Systematic Reviews and Meta-Analyses) checklist.

## Abbreviations

2SLGBTQIAP+: two-spirit, lesbian, gay, bisexual, transgender, queer or questioning, intersex, and asexual
GBMSM: gay, bisexual, and other men who have sex with men
LDA: latent Dirichlet allocation
LGBTQ+: lesbian, gay, bisexual, transgender, and queer
MPXV: mpox virus
MSM: men who have sex with men
PHEIC: public health emergency of international concern
WHO: World Health Organization

## Data Availability

As this article is a scoping review, most of the data generated during this study are included and listed in this published article. A more detailed data set of the studies included in this review is available from the corresponding author on reasonable request.
