# Supplementary figures and images for: Characteristics, Influence, Prevention, and Control Measures of the Mpox Infodemic: Scoping Review of Infodemiology Studies
Source: J Med Internet Res. 2024 Aug 30;26:e54874. doi: 10.2196/54874 (PMC11399743; doi:10.2196/54874)

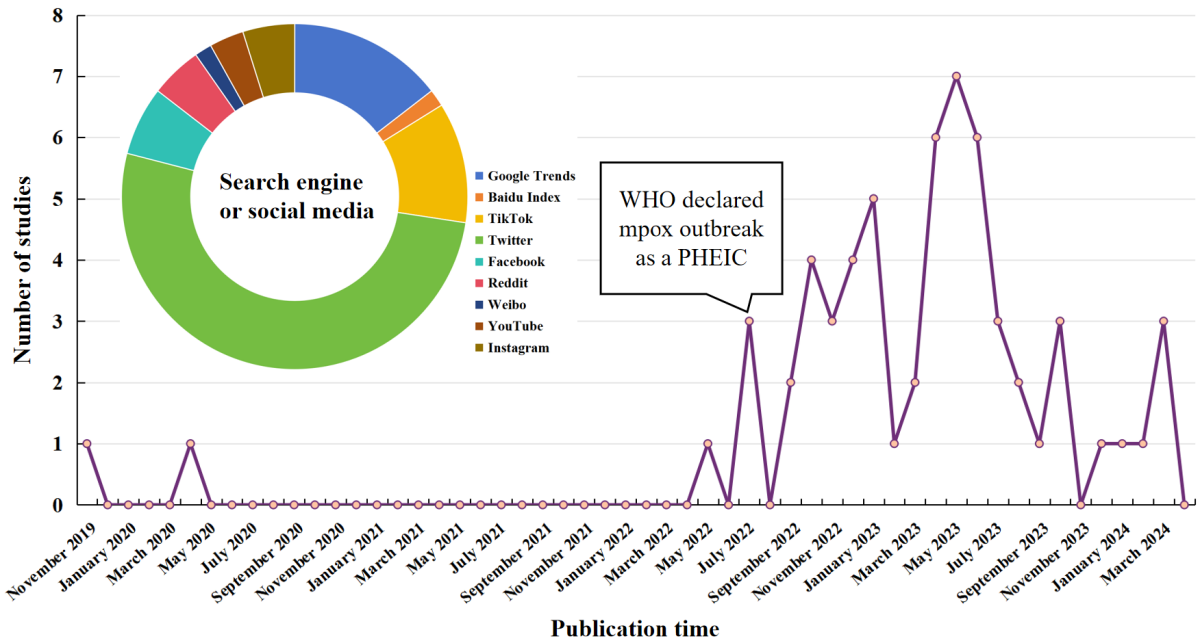

Supplement: Multimedia Appendix 1 [file jmir_v26i1e54874_app1.png]
